# Exploring Essential Issues for Improving Therapeutic Cancer Vaccine Trial Design

**DOI:** 10.3390/cancers12102908

**Published:** 2020-10-10

**Authors:** Constantin N. Baxevanis, Sotirios P. Fortis, Alexandros Ardavanis, Sonia A. Perez

**Affiliations:** 1 Cancer Immunology and Immunotherapy Center, Saint Savas Cancer Hospital, 171 Alexandras Avenue, 11522 Athens, Greece; fortis@ciic.gr (S.P.F.); perez@ciic.gr (S.A.P.); 2 1st Medical Oncology Clinic, Saint Savas Cancer Hospital, 171 Alexandras Avenue, 11522 Athens, Greece; ardavanis@yahoo.com

**Keywords:** cancer vaccines, vaccine formulation, clinical design, biomarkers, AE37 vaccine, delayed clinical effect

## Abstract

**Simple Summary:**

Therapeutic cancer vaccines have failed to demonstrate clinical improvements in phase III clinical trials over the past two decades. This has led to a rather discouraging view regarding their role in the field of cancer immunotherapy. Herein, we critically examine important issues for improving cancer vaccination strategies in an attempt to rekindle interest for this type of immunotherapy. We highlight the importance of proper clinical design in terms of selected groups of patients, taking into consideration (a) changes in initially established standard-of-care treatments; (b) the appropriate follow-up period necessary to achieve meaningful results; (c) statistical considerations for the delay of treatment effects (i.e., time for development of an effective immune response), thus excluding irrelevant early events; and (d) appropriate biomarkers that could guide vaccinations with clinical benefits to patients. Tackling the aforementioned challenges, therapeutic vaccines could take their rightful place in the immunotherapy hall of fame.

**Abstract:**

Therapeutic cancer vaccines have been at the forefront of cancer immunotherapy for more than 20 years, with promising results in phase I and—in some cases—phase II clinical trials, but with failures in large phase III studies. After dozens of clinical studies, only Dendreon’s dendritic cell vaccine Sipuleucel-T has succeeded in receiving US FDA approval for the treatment of metastatic castrate-resistant prostate cancer. Although scientists working on cancer immunotherapy feel that this is an essential breakthrough for the field, they still expect that new vaccine regimens will yield better clinical benefits compared to the four months prolonged median overall survival (OS) Sipuleucel-T demonstrated in the IMPACT phase III clinical trial. Clinical development of cancer vaccines has been unsuccessful due to failures either in randomized phase II or—even worse—phase III trials. Thus, rigorous re-evaluation of these trials is urgently required in order to redefine aspects and optimize the benefits offered by therapeutic cancer vaccines. The scope of this review is to provide to the reader our thoughts on the key challenges in maximizing the therapeutic potentials of cancer vaccines, with a special focus on issues that touch upon clinical trial design.

## 1. Introduction

Therapeutic vaccines have attracted the attention of researchers and oncologists for decades. The idea that they function in a dual fashion—namely, to generate antitumor immunity by priming cancer peptide-specific naïve T cells, thereby increasing the frequencies of tumor-reactive T cells and reinvigorate preexisting antitumor immunity—was attractive enough to induce an excessive enthusiasm in the early years of their application. Nonetheless, the era of cancer vaccines was disappointing because the tempting clinical efficacy seen in preclinical models and early phase clinical trials was missed in large randomized phase II and III trials [1]. These negative results, as opposed to the current successes with immunotherapeutic approaches (including immune checkpoint inhibition and adoptive cell therapy with genetically engineered T cells), have greatly discouraged the use of vaccines for cancer treatment. However, despite these failures, the therapeutic capacity of cancer vaccines is still far from being fully explored. To move the field forward it is essential to learn from the failures of the past and to use this knowledge in order to improve the clinical efficacy of cancer vaccines. 

There are two major issues that need to be addressed in order to gain useful insight towards effective cancer vaccines. The first issue concerns the potential of a vaccine to properly activate patients’ immune systems. On the basis of our knowledge of the mechanistic pathways involved in the generation of efficient antitumor immune responses, we should consider vaccine formulation to be an essential platform on which to undertake improvements that can make vaccines work better. 

The second issue that needs to be examined is the trial design. Along with the issues surrounding the potency of a cancer vaccine, the clinical study design has a central role for both immunologic and clinical outcomes. The disappointing clinical outcomes from large phase III trials in the past have resulted in a rather pessimistic view regarding the future of cancer vaccines. Nowadays, the limitations of those trials have become more apparent, and it is not difficult to see why those initial failures occurred. One important aspect that was missed during the early era of cancer vaccines was that chemotherapies and active immunotherapy attack tumors utilizing different mechanistic pathways, and as a consequence cancers respond differently to these therapeutic approaches. In general lines, tumor cells respond to cytotoxic chemotherapy in an accelerated fashion either by differentiation (which is linked to inhibition of proliferation), arrest of cell division (which is linked to senescence), or cell death (which is caused by DNA damage or inhibition of RNA transcription). In contrast to chemotherapies, therapeutic vaccines primarily target the immune system, which subsequently attacks the tumor. This can be a long process that can take several weeks to achieve complete development, but it results in the generation of a tumor-specific immune memory that slows down tumor cell growth rates via a continuous immune pressure derived from dynamic tumor immune surveillance. Such an effective antitumor immune response may establish a tumor growth equilibrium, resulting in prolonged overall survival (OS). 

It is plausible that potent cancer vaccines have been unfairly discarded because they were applied to the wrong patients, tested with imprecise immunologic assays, or evaluated using the wrong endpoints. Moreover, in many instances there has been a huge gap in the time period between phase II and phase III trials of up to as much as 10 years. During that period, advances made in the standards of care considerably prolonged patients’ survival—difficult to achieve with a vaccine. The scope of our review is not to provide a full report of cancer vaccinations thus far, but to introduce a necessary conceptual platform that has the potential to advance our understanding of the direction in which the field should be moving, focusing specifically on strategies to elicit clinically meaningful responses. We propose refinements of the cancer vaccine study design, and try to advance perspectives.

## 2. Constructing Effective Vaccines for the Therapeutic Treatment of Cancer

### 2.1. Tumor Antigens and Antigen Formulation

The majority of therapeutic cancer vaccines comprise well-defined tumor antigens that can be injected in various formulations—for instance as genetic material (DNA or RNA), or full-length protein or synthetic (poly)peptides from tumor-associated immunogenic proteins [2]. In general, multi-epitope tumor vaccines are preferred, as they overcome the constraints posed by single epitope vaccines while containing multiple defined antigens that may simultaneously induce both CD8+ T cell cytotoxic and CD4+ T cell helper responses, thus supporting the maintenance of long-lasting antitumor immunity [3]. In principle, multi-epitope tumor vaccines limit the risk of immune escape tumor variants, which, under the selective pressure of immunotherapies via genetic or epigenetic modifications, have down-regulated, single-targeted antigens or HLA class I or class II alleles. Recently, there has been a renewed interest in defining antigen vaccines through the use of high-throughput technologies and prediction algorithms, and this has enabled the selection of neoantigen candidates that are likely to induce an antitumor T cell response and can be used as therapeutic cancer vaccines [2]. Another promising approach to generating defined antigen vaccines has grown out of the elution, purification, and sequencing of the peptides from the tumor cell surface via mass spectrometry. These peptides are by definition processed and presented by the HLA molecules of a patient’s tumor [4], and they represent the effective immunopeptidome of a given tumor and provide a personalized vaccine containing a mixture of peptides, some of which might be potent tumor neoepitopes.

Along with the identification and characterization of tumor antigens, there have been extensive attempts to potentiate the antigenicity and immunogenicity of tumor-associated antigens [2]. On this basis, the most effective vaccine formulation included cells with potent antigen-presenting capacities, such as dendritic cells (DCs) [5]. DC vaccines generated in this way are generally safe—with minimal side effects—and have proven to be feasible and effective in some patients. Sipeuleucel-T, the only therapeutic vaccine so far to have received FDA approval, is formulated by DCs loaded with a prostate-cancer-associated antigen coupled to GM-CSF. Other vaccine formulations comprise mixtures of whole allogeneic tumor cells secreting GM-CSF (such as the GVAX vaccine), or that are administered along with immunomodulators (e.g., anti-TGFβ; belagenpumatucel L). Multiple other vaccine formats have been based on tumor cell lysates, peptide mixtures (renal and hepatocellular carcinoma; IMA901), synthetic long peptides (vulvar intraepithelial neoplasia), recombinant proteins (MAGE-A3), and recombinant viral vectors (PROSTVAC) [1]. Polyvalent neoantigen-based vaccines have shown antitumor activity preclinically and have been tested in early clinical trials [6,7,8]. 

### 2.2. Adjuvants and Delivery Systems

Adjuvants are meant to function as substances for delivering antigens slowly and continuously to maximize uptake by DCs, resulting in more efficient antigen presentation and T cell activation. To this aim, water-in-oil emulsions (e.g., incomplete Freund’s adjuvant) were initially developed to stimulate high and long-lasting T cell responses by creating a depot of slow-released antigen at the site of the injection. The next generation adjuvants utilized in clinical vaccination trials promoting inflammatory processes included TLR-agonists, saponins, and GM-CSF. TLR-agonists such as poly-ICLC (TLR3-agonist), MPL (TLR4-agonist), imiquimod (TLR7-agonist), and CpG ODN (TLR9-agonist) have emerged as a class of effective vaccine adjuvants [9,10]. ISCOMATRIX and QS-21 are saponin-based immune adjuvants that have been used in many clinical trials, and therapeutic vaccines containing these adjuvants are under development for various types of cancer [11,12,13]. Based on its immunostimulatory capacity in various preclinical models, GM-CSF co-injected with the antigen in soluble form, or secreted by autologous DCs or allogeneic whole tumor cells, has been used as an adjuvant in various vaccination studies [14].

The co-delivery of tumor antigens and adjuvants using nanoparticles (NPs) may offer significant advantages for cancer vaccines, including specific and targeted delivery, low toxicity, and immune modulatory effects. NPs may include a variety of biomaterials including liposomes, micelles, hydrogels, photothermal materials (gold nanoparticles), inorganic NPs, spray-dried particles, and synthetic high-density NPs [15,16,17,18]. Virosomes are liposomes containing viral proteins that permit fusion with target cells and deliver adjuvants and/or expression of tumor antigens by the target cells [19]. Microneedles are promiscuous delivery adjuvants and proteins or genetic material through the skin barrier inducing potent antitumor responses [20,21].

Notwithstanding the fact that our understanding for the quality of tumor antigens, vaccine formats, and immune adjuvants has considerably increased over time, we need to understand why, in light of all the progress made, therapeutic vaccines for cancer treatment have demonstrated far more losses than triumphs. Merely putting effort into improving vaccine formulations without considering other mandatory issues (e.g., clinical setting and design, endpoints, immunomonitoring, and response criteria) is like missing the forest for the trees. In the following sections we discuss these issues, starting with our randomized phase II trial of the AE37 vaccine in breast cancer (BCa) patients who failed to show clinical benefits among the entire vaccinated patient population vs. the control group, but, interestingly enough, did so in patient subgroups with distinct disease molecular subtypes.

## 3. The AE37 Vaccine Paradigm

The AE37 vaccine was designed as a hybrid vaccine to chemically link a tetramer from the invariant chain of MHC class II molecules (the Ii-key molecule) to the 776–790 segment of the intracellular domain of HER2/neu (AE36). Through this linkage, the AE36 15-mer has a stronger binding affinity with MHC class II molecules, thus prolonging the interaction of the cognate CD4+ T cells with the antigen-presenting cells and, in this way, inducing more potent CD4+ T cell activation. Moreover, AE36 contains in its sequence HLA-class I binding motifs, thus being capable of activating activate CD8+ T cells specifically (in addition to CD4+ T cells) [22].

In a phase I clinical trial, the AE37 vaccine was administered to disease-free, node-negative BCa patients, whereby it was shown to be safe and immunogenic, inducing robust AE37-specific immunity both in vivo and in vitro, measured as delayed-type hypersensitivity (DTH) reactions and proliferative responses, respectively [23]. Next, AE37 was tested in a multicenter randomized single-blinded phase II trial [24,25] that enrolled 304 clinically disease-free, node-positive and high-risk node-negative BCa patients with HER2+ (IHC 1+ to 3+) tumors. High-risk node-negative patients were defined if they had any of following: HER2 overexpressing tumor, ≥T2, grade 3, lymphovascular invasion, estrogen or progesterone receptor negative. It has to be mentioned that this study (NCT00524277) was started in January 2007, before Trastuzumab had become the standard-of-care adjuvant therapy for HER2-overexpressing patients. Toxicities were in their vast majority of grade 1, with none greater than grade 3. Patients in the vaccine group (AE37/GM-CSF) developed significant AE37-specific IFNγ and proliferative responses as well as DTH reactions as compared to the placebo arm (GM-CSF). Aside from the immunological responses, there were no clinical benefits, as there were no significant differences for the entire study population in terms of disease-free survival (DFS) between the vaccine and control arms, and therefore the trial was characterized as negative. However, when patients were subgrouped by clinicopathological parameters, AE37 was associated with improved clinical benefits. This was the case for patient subgroups stratified by stage (advanced; i.e., stage IIb/III), HER2 expression (low expression) or molecular group triple-negative breast cancer (TNBC). The best clinical benefits were obtained in vaccinated advanced-stage/low-HER2 expressors (log rank *p* = 0.039, HR 0.375) and advanced-TNBC patients (*p* = 0.078, HR 0.184). We should mention that these results were obtained despite the small number of per treatment patients enrolled (i.e., patients who received all six primary vaccinations), subgrouped into either advanced-stage/low-HER2 expression (*n* = 78), or advanced-stage/TNBC (*n* = 18) expression (27.6% and 6.4% of the total per treatment patients, respectively) [24]. Therefore, our study clearly shows that AE37 + GM-CSF may significantly reduce recurrence rates in selected patients, suggesting an appropriate patient population for further clinical development of this vaccine. This AE37 phase II randomized study provides a paradigm by which to clarify that a complete appreciation of therapeutic cancer vaccines can only be achieved when tested in clinical settings that best support their aim—that is, to reinforce the endogenous T cell response against tumor antigens—and should not be expected to achieve clinical results in control groups responding better to a certain standard-of-care. Consequently, the data from the AE37 study suggest that BCa patients with HER2-overexpressing tumors exhibit no clinical responses upon vaccinations, which is very likely given that DFS rates in these patients are very low, reflecting the benefit of adjuvant Trastuzumab therapy. For this reason, these patients are less likely to benefit from the vaccine (although they may respond to it), at least in the time frame of a follow-up predicted for this trial (i.e., five years post enrollment), because the majority of them respond successfully to Trastuzumab, thus obscuring vaccine effects on recurrences and making the standard-of-care one potential reason why the HER2-overexpressing AE37-vaccinated BCa patients failed to demonstrate meaningful clinical benefits in this instance. Thus, it is conceivable that AE37 vaccinations could prove to be better in the advanced stage, in cases of low-HER2 expression and, in particular, in individuals with the triple-negative subtype of BCa. There are two reasons for this: first, these patients are not eligible for Trastuzumab as a standard-of-care, and thus lack any beneficial effects from this type of passive immunotherapy; second, because they have a higher risk of recurrence within the five years of the trial.

Another interesting finding from the AE37 phase II trial was that in the subgroups of BCa patients the AE37 vaccine showed clinical efficacy over the placebo (i.e., the Kaplan–Meier survival curves for the two arms separated 12 months following the vaccinations). This may be attributed to the known delayed effect of immunotherapies, including vaccinations, for demonstrating clinical efficacy. Considering the period needed to generate vaccine-induced antitumor immunity followed by clinical responses, the survival of vaccinated patients may not be influenced at all until several months post-administration. In this time frame, during which the survival curves between the vaccine vs. the control groups are not yet separated, a notable number of events may have occurred in the vaccine arm that, if considered in the statistical analyses, will result in a substantial weakening of statistical power. Statistical significance among the vaccine vs. the control arms in BCa subgroups that benefited from the AE37 vaccinations were higher when events that occurred before the separation of survival curves were excluded. Therefore, it is critically important to take into account the prolonged time intervals necessary to observe immunologic and clinical effects for statistical considerations. To this end, the application of modified statistical procedures to specify hazard ratios pre- and post-separation of survival curves [26] will contribute to a better design of clinical trials. Not taking into account the delayed effect during vaccinations may lead to confusing data and decisions that will lead to a premature termination of the clinical study for futility thus missing some beneficial effects from vaccinations which could be seen at later time-points. In the following paragraph we will discuss a number of negative phase III vaccination trials in the context of issues that touch upon clinical design relative to the AE37 trial. 

## 4. Negative Phase III Clinical Vaccine Trials in the Context of Clinical Setting

Considering the important role of cancer vaccines for cancer immune surveillance, efforts should be made to explain why they have failed to reach their primary endpoints, instead of deleting them from the immunotherapeutic anticancer armamentarium. For this, we need to provide reasonable explanations by critically addressing the reasons that could possibly account for these failures, as this will pave the way for implementing cancer vaccines under the best possible conditions. There are notable examples in the literature that show that changes in clinical design and new standard-of-care therapies implemented in phase III trials have led to improvements in the DFS and/or OS in the control groups; however, this was more difficult to achieve in the vaccine group. 

The MAGE-A3/NSCLC phase II trial was a placebo-controlled study designed to assess the clinical benefit of recombinant MAGE-A3 protein and prolong survival in NSCLC patients after surgery [27]. It is important to note that no concomitant chemotherapy was given, because adjuvant chemotherapy was not a standard-of-care during the period of patient enrollment (2002–2004). After a median period of 44 months post-surgery, recurrences were observed in 35% of patients in the MAGE-A3 group and in 43% in the placebo group, providing a trend of increased disease-free interval (DFI) in the vaccine arm. The subsequent phase III trial (MAGRIT) [28], which enrolled NSCLC patients between 2007 and 2012, was designed to also include patients receiving adjuvant chemotherapy postoperatively, because in the meantime adjuvant cisplatin-based chemotherapy was the standard-of-care for patients with stage II–IIIA disease and had shown improved clinical outcomes. In fact, the three-year DFS for the group of patients receiving adjuvant chemotherapy was 60.4%, whereas in the phase II study the DFS in the placebo group (no chemotherapy post-surgery) was approximately 54%. Moreover, the phase II study included patients with stage IB and stage II disease, ≤8 weeks post-surgery, whereas in the MAGRIT study patients with the more advanced disease stage IIIA were included in addition to stages IB and II. Furthermore, in the MAGRIT study, the no-chemotherapy group was randomized at later time points for vaccinations (i.e., 12 weeks post-surgery). Consequently, there were notable differences between the phase II trial and the MAGRIT phase III trial that may have significantly contributed to the absence of vaccine-induced effects on DFS or OS in the MAGRIT trial in patients either with or without concomitant chemotherapy. We may also consider that during the period of the phase III trial, advances made in surgical or radiation therapies positively influenced clinical responses to subsequent adjuvant chemotherapy that were difficult to achieve through the MAGE-A3 vaccine. The MAGRIT trial is a representative example highlighting the crucial role of clinical design in the failure of a vaccine to show clinical efficacy in a phase III trial, despite the fact that the same vaccine under a similar vaccination schedule had demonstrated immunologic and clinical responses in a preceding phase II study. Another example for this is provided by the next phase III trial, which further suggests that vaccine efficacy may be better approached through the selection of appropriate patient subgroups on the basis of molecular biomarkers.

The IMA901 poly-epitope vaccine was used in a randomized phase II trial to vaccinate patients with advanced clear cell renal cell carcinoma (ccRCC) with or without a single dose of cyclophosphamide [29]. Patients who received cyclophosphamide had significantly reduced numbers of circulating Tregs and, among those, immunological responders to any of the IMA901 peptides had significantly prolonged survival. This phase II randomized study was the first to demonstrate an association between cyclophosphamide and immunological responses with clinical efficacy after vaccination. In the subsequent phase III IMPRINT trial, a different clinical design was used for testing IMA901 in patients with metastatic ccRCC. These patients were randomized in two arms to receive either sunitinib alone or in combination with the vaccine. Sunitinib, which had become the standard-of-care as a first line therapy for RCC, was supposed to replace cyclophosphamide for its beneficial immunomodulatory effects, based on a previous report showing a reduction in circulating Tregs (although the reduction was not statistically significant) [30]. There were no differences in survival among the two groups stratified by immunological responses to the vaccine. An interesting finding within the IMPRINT study was the observation of the lower (three-fold) magnitude of CD8+ T cell responses compared with the phase II study, which might have resulted from an insufficient T cell priming induced indirectly by sunitinib via a reduction in the number of monocytes (detectable after the first round treatment with sunitinib). To this end, it is worth mentioning that sunitinib also exerts other effects on monocytes negatively influencing T cell responses like the induction of IL-10 [31]. The other possibility could be the presence of high numbers of Tregs in the absence of cyclophosphamide. However, besides its negative effects on immunologic responses, most striking is the fact that sunitinib—the current standard-of-care for first-line treatment of metastatic ccRCC—has demonstrated proven clinical efficacy, which may have obscured vaccine effects on survival [32]. Interestingly, an additional parameter that could explain the negative results of this trial is that ccRCC consists of four molecular subtypes, of which one poorly responds to sunitinib treatment [33]. Thus, it would had been interesting to test vaccine-induced clinical benefits in patients belonging to this particular subgroup as, in addition, this subtype is associated with an inflammatory, Th1-oriented (albeit suppressive) tumor microenvironment. This suggests that only one subgroup of ccRCC patients had inflamed tumors that could attract IMA901-induced T cells and gain clinical benefits, provided that these cells could resist the hostile tumor microenvironment (e.g., when combined with immune checkpoint inhibitors or other suppressor inhibitors). What we have learned from the IMPRINT phase III trial is that the design was wrong because of the unfortunate choice to include sunitinib. The majority of patients with ccRCC responded better to this standard-of-care, thus obscuring any vaccine-induced effects on OS. Moreover, the exclusion of cyclophosphamide from the vaccine arm resulted in an induction of weak immunological responses, diminishing any chances to observe clinical benefits.

Another example of a negative phase III trial that supports the notion that the available standard therapeutic treatments may confound vaccine-induced survival results was provided through the PROSPECT study [34]. In this phase III study, PROSTVAC—a recombinant virus vaccine targeting PSA and three co-stimulatory molecules—was used to vaccinate asymptomatic or minimally symptomatic metastatic castration-resistant prostate cancer (mCRPC) patients. The PROSTVAC vaccination schedule consists of a priming vaccination with the recombinant vaccinia virus PROSTVAC, followed by booster vaccinations with the fowlpox virus PROSTVAC. While the PROSPECT study did not meet its primary endpoint (OS), PROSTVAC induced a longer median OS by 8.5 months (25.1 vs. 16.6 months for controls). The phase II trial recruited patients during the period between 2003 and 2005, during which docetaxel was the only standard-of-care. On the other hand, until the PROSPECT study was completed (2015), a range of new efficient survival-prolonging treatments became available for men with mCRPC, including androgen-directed therapies (e.g., enzalutamide and abiraterone). Because of this, the median OS in the placebo arm in the PROSPECT study was two-fold higher (up to almost three years) compared to that from the phase II trial. Generally speaking, the role of vaccine-based immunotherapy, which has traditionally been the standard-of-care in prostate cancer, will be greatly challenged in the future, considering the many effective drugs improving OS in this type of cancer. It is conceivable that these life-extending therapies have negatively influenced the possibility of achieving any vaccine-induced clinical efficacy within the time frame of this phase III trial. The data from the PROSPECT trial again place emphasis on the important role of effective therapies used as the standard-of-care. These may mask vaccine efficacy when comparing groups of patients receiving the standard-of-care plus the vaccine vs. the standard-of-care alone, and therefore should not be overlooked when designing vaccine phase III trials. 

Another notable example in this direction is the Neuvax vaccine in the phase III PRESENT trial. Neuvax is a vaccine consisting of the E75 peptide (the HER2 nonamer: 369–377) and GM-CSF as an adjuvant, and was used to vaccinate in phase I and phase II trials (i.e., stage II or stage III breast cancer patients who expressed HER2 at all levels (immunohistochemical staining 1+, 2+, 3+)). In the primary analysis, with a median 20 months follow-up after vaccination, the recurrence rates in the vaccine arm were 5.6% vs. 14.2% in control arm (*p* = 0.04) [35]. Thus, the studies met their primary endpoint and opened the way for the PRESENT phase III trial, which was terminated at interim analysis, due to no significant differences in DFS between the vaccinated vs. placebo groups [36]. In contrast with the previous phase I/II trials, assessment of recurrences in the phase III study did not follow the standard recommendations for clinical follow-up, according to which no CT imaging tests are necessary in the lack of clinical signs [37]. Instead, protocol-specified annual CT scans were mandatory in the PRESENT trial, although without biopsy verification (in cases of suspicion of a recurrence). The PRESENT study demonstrates another failure of a phase III vaccination trial resulting from improvements to the standard-of-care, and most importantly due to an unconventional assessment of recurrences. Thus, in the PRESENT study, there were more recurrences in the vaccine group (9.8% vs. 6.3% for the control arm), although the phase I and phase II trials included patients at almost same risk of recurrence with the PRESENT trial. This decrease of more than two-fold in the recurrence rates of the control groups in the phase I/II vs. the phase III trials may have been due to the fact that taxanes were added in the standard-of-care for adjuvant chemotherapy protocols by the time the PRESENT study was recruiting patients (2011–2015), whereas taxanes were not included in standard practice 10 years earlier when the phase I and phase II studies started. It is also worth mentioning that the number of recurrence events found by protocol-specified imaging in the vaccine arm were higher than in the placebo arm (54.1% vs. 29.2%), whereas the percentage diagnosed clinically in the vaccine arm, as per standard-of-care, was lower than in the placebo arm (45.9% vs. 70.8%), which is in line with the findings of the early stage trials. As the authors proposed, the increased recurrence rates in the vaccinated patients could have been due to pseudoprogression (PP), a phenomenon of immunotherapy reported in melanoma patients [38,39] that describes a situation in which tumor burden is increased not because of tumor progression, but due to immune cell infiltration, or because new lesions appear as early signs of antitumor immunologic responses. In a similar fashion, in the PRESENT study pseudoprogression may also have occurred in breast cancer patients with minimal residual disease in the adjuvant setting as an early indication of antitumor immune reactivity. However, this was never confirmed, because serial imaging was not included for deciding if recurrences reflected objective tumor progression or pseudoprogression as per iRECIST criteria [40]. 

## 5. Predictive Biomarkers

From all the above, it becomes clear that a complete appreciation of vaccination-based cancer therapy can be only obtained in trials extensively supporting their purpose, which is to reinvigorate endogenous memory antitumor immunity and through this to produce meaningful clinical efficacy. Although this was intentionally attempted in all the above-mentioned phase II/III trials, vaccines still failed to demonstrate clinical benefits because their possible therapeutic capacities was hidden either by selecting an inappropriate group of patients (which in certain cases was even not included in the preceded studies), as a result of more effective therapies being applied as the standard-of-care and/or stemming from other improvements in the standard-of-care such as CT- or PET-imaging for clinical follow-up. These life-extending therapies significantly lowered any differences in survival between the vaccine vs. the placebo groups, at least within the defined time frame of the clinical study; however, the selection of patient subgroups who might benefit from vaccination opens a strong possibility of uncovering vaccine-mediated clinical effects, as this was shown in the AE37 trial and suggested by the IMPRINT trial. Because cancer vaccine therapies specifically target tumor antigens, we should recognize that only a certain number of cancer patients will benefit from those. Consequently, in selected patient groups, vaccination strategies could induce substantial clinical benefits, and therefore patient selection is the key to assessing vaccine efficacy. To this end, predictive/surrogate biomarkers informing the early indication of response or predicting clinical benefits are a priority. Presently, there are no validated biomarkers, but nevertheless there is a variety of immune responses whose assessment at baseline and during or after initial vaccinations might be valuable to predicting clinical responses in the long term. In this respect, we have provided valuable information from the AE37 phase II trial by demonstrating clinical benefits in patients responding to the vaccine in vivo and in vitro in the form of DTH and ELISPOT-based IFNγ production, respectively. Nevertheless, the exact time point that could give meaningful predictive information using these immune responses (e.g., pre-vaccination, after a number of vaccine doses or at the end of the active phase of the trial) has not been clearly defined. What we can clearly see is that patients with high local reactions within 48 h after the first vaccine administration (reflecting high levels of preexisting immunity to AE37) have also had significant clinical responses [41]. However, preexisting immunity alone may not always correlate with vaccine-related clinical outcomes, as many factors characterizing specific tumors (e.g., HLA loss, antigen loss, hostile tumor/micrometastasis microenvironment, T cell exhaustion, etc.) may negatively affect the efficacy of the specific antitumor preexisting immunity. Thus, no single immune biomarker will prove sufficient, and biosignatures that incorporate multiple predictive biomarkers will be needed for each cancer type and for each therapeutic vaccination setting.

Data from integrated “omics” (including genomic, transcriptomic and proteomic) analyses combined with immunological surrogates (ex vivo multicolor analyses, in vitro and in vivo assessment of immunological function) could function in the form of combined biomarkers for clinical response measures. However, irrespective of whether biomarkers consist of one or multiple parameters, we feel that these biomarkers should provide a reliable view of patients’ immune systems at baseline, during vaccination and over the long term during booster injections, so that combined with clinical follow-up they can predict clinical outcomes. Following retrospective detection, prospective validation of predictive biomarkers will require clinical studies with well-defined patient cohorts and primary endpoints. Although we are not yet at the point of having discovered the ideal biomarkers, further efforts to appreciate their reproducibility and sensitivity will have a valuable impact on their clinical importance with better clinical outcomes in selected groups of patients.

## 6. The Delayed Onset of Benefit during Vaccinations

As discussed above, the data from the AE37 phase II trial demonstrated that vaccinating BCa patients in the adjuvant setting can be successful in certain subgroups and can also significantly prevent extended recurrences. In the same study, it was also clear that the Kaplan–Meier survival curves for the vaccine vs. the control groups separated at later time points, which is in line with other immunotherapy trials. This delayed effect is justified by the mechanism of action of cancer vaccines that first need a period to activate the immune system before exerting antitumor effects, followed by clinical responses. Consequently, patients who recur at earlier time points will have high tumor burdens by the time the immune system develops antitumor reactivity, and thus will have less chances to benefit from vaccination. Because of the vaccines’ delayed therapeutic effects when designing vaccine clinical trials, statistical planning should consider that the vaccination protocol may not be capable of preventing early recurrences. Hence, modified statistical models will be required for sample and power calculation to properly and efficiently incorporate the delayed effect in an effort to improve the design of randomized phase II and III clinical trials [42].

Another point that comes up because of the delayed therapeutic effect of cancer vaccines is that some patients seem to progress after treatment initiation and, in several occasions, continue to progress before they finally respond to immunotherapeutic treatment. Such delayed clinical responses to immunotherapies have led to the conclusion that clinical outcomes in the course of immunotherapeutic modalities would be better appreciated by the immune-related response criteria (irRC), instead of RECIST, which would also assist in finding better treatment options for patients [40]. The above described situation characterized by an initial disease progression (PD) followed by objective responses is called PP (see Section 4), and is usually attributed to a heavy infiltration of tumor lesions by activated cytotoxic T lymphocytes that initially cause an enlargement in tumors/micrometastases, then shrink. Thus, to properly judge clinical responses during immunotherapies, it will be useful to consider that the time frame for their detection may be extensively longer than that required during chemotherapy. PP has been described in the context of trials using immune checkpoint inhibitors, but also in other immunotherapeutic trials including vaccines. This issue was raised in the PRESENT phase III trial (discussed in Section 4) to explain the increased frequency of recurrences in the vaccine arm vs. the placebo. In this trial, the lack of biopsy confirmation and of serial imaging made discrimination between PP and recurrence uncertain. The exploration of biological mechanisms and the discovery of novel biomarkers to differentiate between PP and PD is a challenging issue because it will assist the improvement of irRC. The late separation of survival curves as well as the unusual patterns of response—both of which have been observed due to the delayed effect of vaccinations—should be taken into consideration when planning interim analyses at early time points because this may overlook a delayed clinical benefit.

## 7. Conclusions

Currently, the vast majority of therapeutic cancer vaccines that have been employed in large phase III trials have failed to reveal clinical benefits for the vaccinated patients. Should we consider this failure to be a result of the reduced capacity of the vaccine formulation to induce potent immunological responses that could be translated into meaningful clinical responses? Or is it possible that the number of clinical responders among the total enrolled patients is too low to permit statistical comparisons? Given that the same vaccine formulations have been successfully tested in preceding phase II trials, these two possibilities seem rather unlikely. Certainly, we should not overlook the issue of the immunogenicity of the vaccine, keeping in mind that this greatly depends on the generation of an inflammatory milieux at the vaccine site to allow trafficking of DCs followed by uptake and processing the tumor antigens to be presented to the T cells in the draining lymph nodes. To this end, as also discussed above, synthetic NP-based biomaterials may offer significant advantages to cancer vaccines through the co-delivery of tumor antigens and adjuvants. Moreover, a full recognition of cancer vaccines can be obtained when these are tested in clinical settings that appropriately address their aim: to reinforce the antitumor T cell immunity. Strategies combining multiepitope vaccines with immune checkpoint inhibition will be mandatory to maintain effective immune surveillance and prevent tumor escape [43]. In addition, complementary treatments to block immune suppression in the tumor microenvironment, including depletion of regulatory T cells, inhibition of suppressor enzyme (e.g., TGFβ, IDO) function or blocking metabolic reprogramming, is mandatory to realize the full capacity of therapeutic cancer vaccines [2,44,45]. One important issue to consider when applying combinatorial modalities to strengthen antitumor immunity is the release of IFNγ by the activated T cells. Upon exposure to IFNγ, tumor cells will develop adaptive immune resistance against the immune attack via expression of various ligands for immune checkpoints [46]. This needs to be restrained in order to allow the vaccine-induced T cells to control tumor growth for extended periods of time, thus generating durable antitumor immunity. Chemotherapy—induced immunogenic cell death eliciting adaptive antitumor immunity [47]—is an interesting aspect to consider when designing protocols based on therapy combinations including chemotherapy and vaccines.

In this review, we intended to place emphasis on a third possibility that could account for the negative results in phase III vaccine trials, namely, the clinical design. It is noteworthy that moving to a phase III from a preceding phase II trial usually takes a long time, during which improvements in the standard-of-care may have been made. Such improvements will advance clinical responses among non-vaccinated patients in the phase III trials, thus prolonging their survival and possibly masking any vaccine effects within the defined time frame of the clinical study. Consequently, phase II trials should be organized in a sufficiently sensitive manner in order to demonstrate the best information for clinical benefits for moving to phase III studies in time intervals securing identical standards-of-care. The standard-of-care for clinical vaccine trials should also be considered interconnected with patient selection. For instance, in the IMPACT phase III trial, Sipuleucel-T, the only FDA-approved therapeutic vaccine, prolonged OS in prostate cancer patients who had asymptomatic metastatic castrate-resistant disease (mCRPC), and during vaccinations did not need to concomitantly receive docetaxel. Thus, the vaccine group was compared to a placebo. Recruiting patients with asymptomatic mCRPC—a rather slow progressing disease—to receive a vaccine in the absence of any other treatments was an appropriately designed clinical trial that allowed the vaccine to unleash its full potential. In contrast, two other phase III trials (VITAL 1 and 2) failed because they enrolled patients with more advanced symptomatic mCRPC who were on docetaxel as the standard-of-care, which prolonged patients’ OS to an extent that could not be achieved by the vaccine (GVAX). By applying a vaccine to patients with clinical features that predispose failure (e.g., high tumor load, aggressive disease types) will ultimately result to the failure of the vaccine. 

A proper clinical vaccine design should also consider the delayed time needed for translating vaccine-induced antitumor immune reactivity to clinical efficacy. Because of this delay, patients’ clinical courses may not be affected for several months post-vaccination. Hence, the effective period for a vaccine should start when the first indications of clinical responses among the vaccinated patients are detectable, which is delimited by the time the survival curves separate. This situation reduces the ability for statistical power to differentiate between the vaccine vs. no-vaccine groups because a sizeable number of events can happen in the time period before separation of the survival curves. Therefore, it is important to perform statistical analyses that will adequately calculate sample size and power, efficiently incorporating clinical efficacy delay. This may be appropriate for providing additional information about the time plan for interim analyses in subsequent phase III trials in the sense that these will be done at the right time points to avoid misleading conclusions that could be detrimental to an otherwise successful trial. 

**Figure 1:**
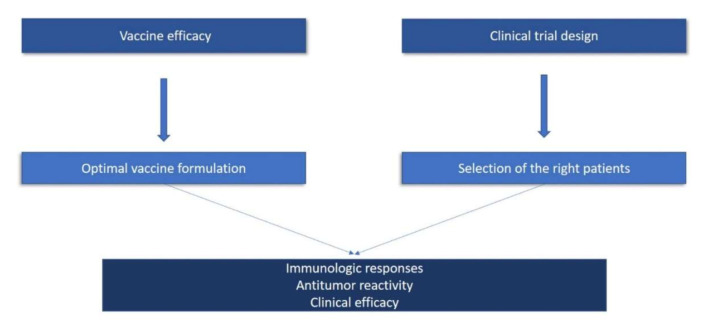
There are two major pillars that determine the potency of therapeutic cancer vaccines. The first pillar deals with issues aimed at increasing vaccine efficacy by optimizing vaccine formulation. The second pillar addresses issues of the proper design of clinical trials that ultimately lead to the selection of the appropriate patient group to vaccinate. The successful use of these two pillars will result in robust antitumor immunity that will translate to antitumor reactivity and meaningful clinical efficacy.

**Figure 2:**
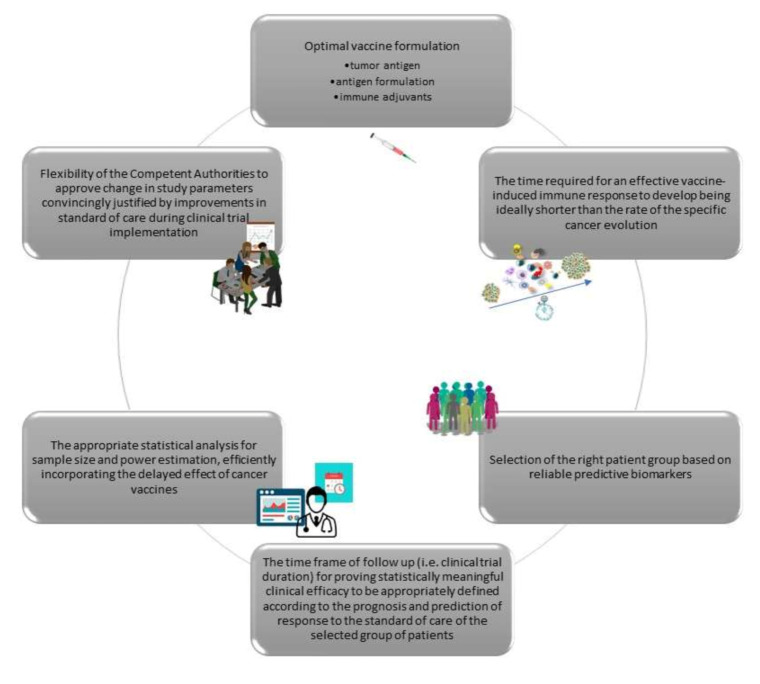
Clinical cancer trials utilizing cancer vaccines report vaccine-induced immunity in the context of different clinical outcomes. Based on our knowledge of why therapeutic cancer vaccines have produced negative results, we propose six key issues that should be critically examined in order to make vaccines work better. These include: (a) the vaccine formulation; (b) the delayed therapeutic effects during vaccinations that should be considered while planning a vaccine trial; (c) the discovery of reliable biomarkers to guide the selection of patients most likely to benefit clinically; (d) the duration of a clinical trial to allow the detection of vaccine-mediated substantial clinical efficacy considering the standard-of-care of the vaccinated patients; (e) adequate statistical models; and (f) the role of regulatory agencies to better assist the clinical development of cancer vaccines.

To summarize, based on lessons learned from previous cancer vaccine studies, there are certain important issues that need to be critically addressed in order to develop effective cancer vaccination immunotherapies. Generally speaking, these comprise two groups: one that examines the parameters that are critical for vaccine efficacy, and one that considers the issues in properly designing vaccination trials (Figure 1). In particular, we have discussed thematic areas exploring (i) the formulation of the vaccine; (ii) the time required for an effective vaccine-induced immune response to develop (which should be significantly shorter than the rate of the specific cancer progression); (iii) the selection of the patient group that may have the best benefit from the vaccine based on reliable predictive biomarkers; (iv) the time frame of follow up (i.e., clinical trial duration) for allowing statistically meaningful clinical efficacy to be appropriately defined according to the prognosis and prediction of response to the standard-of-care for the selected group of patients; (v) an appropriate statistical analysis of sample size and power estimation, efficiently incorporating the delayed effect of cancer vaccines; and (vi) flexibility of the competent authorities to approve changes in study parameters justified convincingly by improvements in standard-of-care during clinical trial implementation (e.g., either by restricting the group of evaluable patients or the duration of the trial according to the new conditions, or even by changing the nature of the trial from a superiority to a non-inferiority study), without violating good clinical practice (Figure 2). 

These important issues should be considered for future evaluations in order to have a holistic view of the best approach for a well-designed clinical protocol to support the full potential of immune system response to the vaccine, and to turn this effectively against a patient’s tumor. The clinical development of cancer vaccines will be more successful on these grounds, leading to an ambitious view of their potential within the immunotherapy field.
